# Poly (I:C)-Potentiated Vaccination Enhances T Cell Response in Olive Flounder (*Paralichthys olivaceus*) Providing Protection against Viral Hemorrhagic Septicemia Virus (VHSV)

**DOI:** 10.3390/vaccines9050482

**Published:** 2021-05-10

**Authors:** Jin Hong Chun, Jae Wook Jung, Young Rim Kim, Jassy Mary S. Lazarte, Si Won Kim, Jaesung Kim, Kim D. Thompson, Hyoung Jun Kim, Tae Sung Jung

**Affiliations:** 1 Laboratory of Aquatic Animal Diseases, Research Institute of Natural Science, College of Veterinary Medicine, Gyeongsang National University, 501-201, 501, Jinju-daero, Jinju-si 52828, Korea; hilanamang@naver.com (J.H.C.); wjdwodnr0605@gmail.com (J.W.J.); yl0808@nate.com (Y.R.K.); jassylazarte@yahoo.com (J.M.S.L.); ksw0017@hanmail.net (S.W.K.); afteru70@gmail.com (J.K.); 2 Moredun Research Institute, Pentlands Science Park, Bush Loan, Penicuik, Midlothian EH26 0PZ, UK; Kim.Thompson@moredun.ac.uk; 3 Pathology Research Division, OIE Reference Laboratory for Viral Haemorrhagic Septicaemia (VHS), National Institute of Fisheries Science, 216, Gijanghaean-ro, Gijang-eup, Busan 46083, Korea; hjkim1882@korea.kr; 4 Centre for Marine Bioproducts Development, Flinders University, Bedford Park, SA 5042, Australia

**Keywords:** viral haemorrhagic septicaemia (VHS), poly (I:C), vaccination, T cell responses, flow cytometry, olive flounder (*Paralichthys olivaceus*)

## Abstract

Viral hemorrhagic septicemia (VHS), caused by viral hemorrhagic septicemia virus (VHSV), is a viral disease affecting teleosts, and is the major cause of virus-related deaths in olive flounder (*Paralichthys olivaceus*). Research has focused on ways to control VHS, and recently, the use of polyinosinic-polycytidylic acid poly (I:C)-potentiated vaccination has been investigated, whereby fish are injected with poly (I:C) and then with live pathogenic virus, resulting in a significant decrease in VHSV-related mortality. T cell responses were investigated in the present study after vaccinating olive flounder with poly (I:C)-potentiated vaccination to understand the ability of poly (I:C) to induce T cell immunity. Stimulation of T cell responses with the poly (I:C)-potentiated vaccination was confirmed by examining levels of CD3^+^ T cells, CD4-1^+^ T cells and CD4-2^+^ T cells. Higher levels of CD4-2^+^ T cells were found in vaccinated fish than CD4-1^+^ T cells, believed to result from a synergistic effect between poly (I:C) administration and pathogenic VHSV immunization. More importantly, the role of CD4-2^+^ T cells in the antiviral response was clearly evident. The results of this study suggest that the outstanding protection obtained with the poly (I:C)-potentiated vaccination is due to the robust immune response initiated by the CD4-2^+^ T cells.

## 1. Introduction

Viral hemorrhagic septicemia (VHS) is caused by viral hemorrhagic septicemia virus (VHSV), belonging to the genus Novirhabdovirus, within the family Rhabdoviridae. The virus consists of a negative-sense, single-stranded RNA genome of approximately 11,000 nucleotides [1]. VHS outbreaks have occurred in the northern hemisphere since 1965 [2,3,4] but have also been reported in farmed olive flounder (*Paralichthys olivaceus*) in South Korea since 2001, where VHS is the biggest cause of virus-related deaths and is responsible for 12.5% of all olive flounder mortalities in Korean aquaculture [5,6,7,8]. Olive flounder production was worth 443 million USD (37,238 tons) in 2018, representing half of all South Korean aquaculture production [7,8]. Different kinds of vaccines have been developed to address the devastating effect of VHS on the global aquaculture industry, including DNA and formalin-based vaccines [9,10,11]. DNA vaccines are considered the most effective under experimental conditions, but there have been concerns about their long-term safety, such as the use of genetically modified organisms (GMO) in fish farmed for human consumption [11]. On the other hand, formalin-treated VHSV vaccines require relatively high doses of the virus to trigger a protective response, and they have been expensive to develop [12].

Recently, the use of polyinosinic-polycytidylic acid (poly (I:C))-potentiated vaccination, involving the simultaneous injection of poly (I:C) with live pathogenic VHSV, has provided significant protection against VHS in olive flounder [13,14,15]. Poly (I:C)-potentiated vaccination has also been tested against infectious hematopoietic necrosis (IHN) in rainbow trout (*Oncorhynchus mykiss*) and viral nervous necrosis (VNN) in seven-band grouper (*Epinephelus septemfasciatus*) [14,16,17,18]. Poly (I:C), a synthetic dsRNA, is an analog of viral dsRNA that induces a transient, non-specific antiviral effect in fish [14,17,19,20]. Unlike DNA-based vaccines, poly (I:C) is an unstable RNA molecule and does not persist to the same extent in fish tissues. This has helped alleviate consumers’ concerns about using this product in fish farmed for food [14,15,17,19]. Poly (I:C)-potentiated vaccination has been demonstrated to be more effective in generating an immune response than formalin-inactivated VHSV vaccines in olive flounder [13]. However, despite the outstanding efficacy of poly (I:C)-potentiated vaccination against viral diseases in teleosts, little is known about the immune response it elicits in fish.

There are indications of improved antibody responses resulting from the poly (I:C)-potentiated vaccination for a variety of pathogens, measured by enzyme-linked immunosorbent assays (ELISA) [13,16,17,18]. Interestingly, while fish vaccinated with poly (I:C)-potentiated or formalin-inactivated VHSV vaccines had similar serum antibody titers, the poly (I:C)-VHSV group had 100% survival as to the formalin-inactivated VHSV vaccinated group, which only showed 8% survival after infecting vaccinated fish with the virus [13]. This, together with other studies, suggest that evaluation of antigen immunogenicity through antigen-antibody interaction does not necessarily reflect the protective response of the vaccine, and a variety of factors can affect the titer of an antibody response obtained [21]. It is difficult to generalize about the antibody interactions that occur in fish since they have a different and more variable immune response than mammals [22,23]. Instead of using antigen-antibody interactions as a way to evaluate the immunogenicity of a vaccine, a better alternative may be to assess T cell populations using flow cytometry [24]. This is now commonly used to examine T cell responses to a vaccine [24] and is an ideal method for evaluating the ability of poly (I:C)-potentiated vaccines to stimulate T cell immunity.

As with humans and other mammals, lymphocytes in teleosts, including T and B cells, play a key role in adaptive immunity [25]. The response of T cells is particularly important in antiviral immune responses since some vaccines targeting antibody responses provide poor levels of protection against infectious rhabdoviruses [21]. For olive flounder, T cell subsets have been characterized based on a cluster of differentiation (CD) markers, namely CD3^+^, CD4-1^+^, CD4-2^+^, and CD8^+^ T cells [25,26,27,28,29,30]. The CD3 molecule forms a complex with the T cell receptor (TCR) to generate an activation signal in both CD4^+^ and CD8^+^ T cells. The CD4^+^ T cells are subdivided into CD4-1^+^ T cells (T helper 2 cells, Th2) and CD4-2^+^ T cells (T helper 1 cells, Th1) [31]. CD4-1^+^ T cells (Th2) help B cells to produce antibodies and are involved in protecting against persistent antigens [32,33,34]. Signaling of CD4-2^+^ T cells (Th1), on the other hand, helps to activate CD8^+^ T cells, which are important for immunity against intracellular pathogens such as viruses and also activate macrophages and natural killer (NK) cells involved in antigen elimination [33,34,35,36]. In previous studies, we developed monoclonal antibodies (mAb) against CD markers for olive flounder CD3^+^, CD4-1^+^, and CD4-2^+^ [26,27,28]. Similar mAbs have been used in flow cytometry to evaluate T cell populations to establish whether poly (I:C)-potentiated vaccination can indeed initiate adaptive immune responses in vaccinated animals [24,37,38,39].

The aim of this study was to investigate T cell responses to poly (I:C)-potentiated vaccination for VHSV by flow cytometry using the CD marker mAbs we produced previously against CD3^+^, CD4-1^+^, and CD4-2^+^ cells. Knowing which of these cells are involved in response to poly (I:C)-potentiated vaccination is important for the development of effective therapeutics for treating viral diseases in aquaculture such as VHS.

## 2. Materials and Methods

### 2.1. Fish and Viruses

Clinically healthy olive flounders (*Paralichthys olivaceus*; body weight, 62.3 ± 9.6 g) were purchased from Jawook fish farm, located in Muan County, South Korea, and were acclimatized in experimental tanks (temperature 16 °C) at Gyeongsang National University for two weeks prior to commencing experimental work. Fish were screened to ensure they were free of VHSV or other viral infections affecting olive flounder before commencing the study. After acclimatization, each group of fish was distributed to five 200 L plastic tanks (*n* = 30 fish), each receiving 24-cycles of UV-sterilized seawater per day, and were fed a daily diet of dry food pellets. Administration of all treatments was performed after anesthetizing fish by immersing them in tricaine methanesulfonate (MS-222) dissolved in seawater (100 mg/L). Fish were sacrificed by immersing their gills in MS-222 dissolved in tank water (500 mg/mL) prior to any sample collection (Sigma, St. Louis, MO, USA). Three fish per experimental group were sacrificed on each sampling day. All experiments were conducted in accordance with the guidelines on animal ethics.

Epithelioma papulosum cyprinid (EPC) cells (ATCC CRL-2872) were maintained at 15 °C cultured in Eagle’s minimum essential medium (MEM_10_) (containing Eagle’s MEM (Gibco^®^, Invitrogen Co., Carlsbad, CA, USA) supplemented with 10% fetal bovine serum (FBS, Gibco^®^, Invitrogen Co., Carlsbad, CA, USA), 100 units/mL penicillin, 100 μg/mL streptomycin, and 250 ng/mL amphotericin B). For the preparation of virus stocks, confluent EPC cells were infected with VHSV (KJ2008) [13] at a multiplicity of infection (MOI) of 1. After 1 h, the inoculum was replaced with MEM medium containing 2% FBS (MEM_2_), and the cells were incubated at 15 °C until a cytopathic effect (CPE) was observed. When an extensive CPE was evident, the cell medium was collected, cellular debris was removed by low-speed centrifugation, and the concentration of the virus in the cell supernatant was determined by calculating the 50% Tissue Culture Infective Dose (TCID_50_).

### 2.2. VHSV Challenge Test

**Figure 1:**
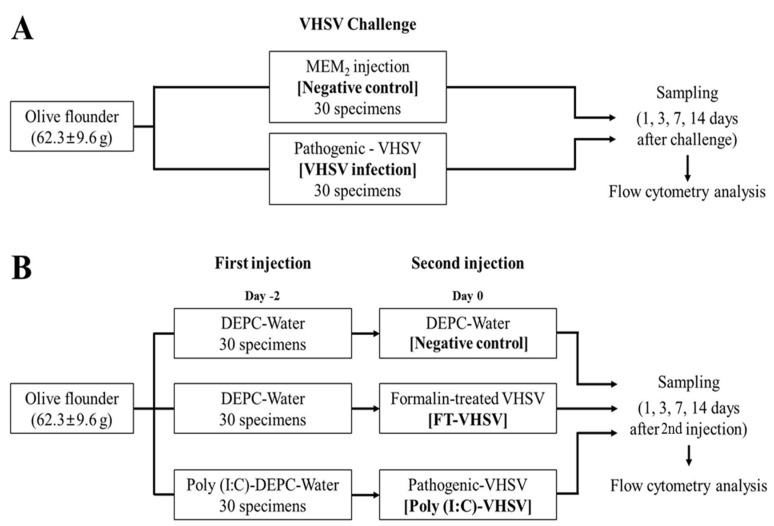
The experimental design of VHSV challenge and vaccination trials. (**A**) Set-up of VHSV infection, VHSV was injected into fish in the VHSV infection group (*n* = 30), while MEM_2_ was injected into fish in the negative control group (*n* = 30). (**B**) In the vaccination experiment, fish in the (Poly (I:C)-VHSV) group were injected with Poly (I:C) two days before they were inoculated intraperitoneally with live VHSV at 0 dpv (*n* = 30). Fish in the FT-VHSV group were injected with DEPC-treated water two days before inoculation intraperitoneally of formalin-treated VHSV (*n* = 30). The negative control group was injected with DEPC-treated water at both −2 dpv and 0 dpv (*n* = 30).

Olive flounders (body weight, 62.3 ± 9.6 g) were separated into two groups (*n* = 30/group). The VHSV-challenged group (VHSV infection) was intraperitoneally infected with 1 × 10^7^ TCID_50_/mL VHSV particles suspended in MEM_2_. For the negative control group (negative control), the fish were intraperitoneally injected with the same volume of MEM_2_ only. The design of the VHSV challenge trial is graphically presented in Figure 1A. The lethal dose of VHSV was determined from the level of survival in both the VHSV infection and negative control groups.

### 2.3. RNA Extraction and RT-PCR for VHSV

Extraction of RNA from spleens of sampled fish was performed using an easy-BLUE Total RNA Extraction Kit (Intron, Sungnam, Korea), then reverse transcribed into cDNA using a TOPscript cDNA Synthesis Kit (Enzynomics, Daejeon, Korea) with Oligo (dT) primers. Extracted RNA was measured with NanoDrop 1000 using a UV-Vis spectrophotometer (Thermo Scientific, Waltham, MA, USA), and then 1 µg was used for reverse transcription. The specific primers for the VHSV RT-PCR were VHSV-FOR (3′-ATCATCCATCTTCCGTTATC-5′) and VHSV-REV (3′-TGTCACCTTGCATGCCATTG-5′). For the RT-PCR cocktail, 2 mL of cDNA template and 10 pM of each primer were used together with AccuPowerProFi Taq PCR premix (Bioneer, Daejeon, Korea). The PCR conditions were as follows: one cycle of initial denaturation at 95 °Cfor 3 min, 35 cycles of denaturation at 95 °C for 30 s, annealing at 60 °C for 30 s, and extension at 72 °C for 1 min. The amplified products were electrophoresed on 2% agarose gels and visualized using Red Safe nucleic staining solution (Intron, Sungnam, Korea). SiZer™-1000 DNA Marker Solution (CAS# 24074, IntronBio, Sungnam, Korea) was used for size estimation by loading 5 μL DNA ladder mixture on the gel. Images were photographed with an AE-9000 E-graph system (ATTO Corporation, Tokyo, Japan).

### 2.4. Preparation for Vaccination

Poly (I:C) (CAS# 42424-50-0, Sigma, USA) was dissolved in diethylpyrocarbonate (DEPC)-treated water (CAS# 7732-18-5, Sigma, USA) at a concentration of 1 mg/mL immediately before use. VHSV was suspended in MEM_2_ at 1 × 10^7^ TCID_50_/mL the previous day and stored at 4 °C. Formalin-inactivated VHSV was prepared as previously described [13]. Briefly, the virus was suspended at 1 × 10^7^ TCID_50_/mL in MEM_2_, followed by the addition of formalin (0.1% *v*/*v*) and briefly mixed and incubated at 4 °C for 5 days without shaking.

### 2.5. Vaccination

Olive flounders (body weight, 62.3 ± 9.6 g) were separated into two groups (*n*_1_ = 30 and *n*_2_ = 60/group). Poly (I:C) (CAS# 42424-50-0, Sigma, St. Louis, MO, USA) was injected intraperitoneally at 100 µg 100µL^−1^ fish^−1^ for the experimental group, while for the negative control group, 100 µL^−1^ fish^−1^ of DEPC-treated water was injected intraperitoneally. Two days post-poly (I:C) injection, 1 × 10^7^ TCID_50_/mL VHSV 100 µL^−^^1^ fish^−^^1^ was injected intraperitoneally into fish in the poly (I:C) group. This group is referred to as the poly (I:C)-potentiated vaccination (Poly (I:C)-VHSV) group. Fish injected with DEPC-treated water were divided into 2 groups (*n* = 30/group), then intraperitoneally injected with either 100 µL^−^^1^ fish^−^^1^ of formalin-inactivated VHSV vaccine (FT-VHSV) or the same volume of DEPC-treated water (negative control). The schematic flow of the vaccination experiment is graphically presented in Figure 1B.

### 2.6. Preparation of Olive Flounder Leukocytes

Leukocytes were isolated from the kidney, liver, peripheral blood (PBL), and spleen of healthy olive flounders (body weight, 62.3 ± 9.6 g; Jaewook fish farm, Muan, Korea) using a Percoll gradient. The kidney, liver, and spleen were removed and homogenized in Dulbecco’s Modified Eagle’s medium (DMEM; Thermo Fisher Scientific, Waltham, MA, USA) with heparin added and then samples were filtered separately using cell strainers (BD Falcon, San Jose, CA, USA), washing through with 10 mL of DMEM. PBL was collected via a syringe containing heparin of 10 IU/mL (Choongwae, Seoul, Korea) and isolated by immediately diluting blood with cold DMEM containing heparin (10 IU/mL) (1:1 dilution). The diluted samples were mixed with 51% Percoll solution (5% 20× phosphate-buffered saline (PBS), 44% distilled water, and 51% Percoll). Leukocytes from kidney, liver, and spleen and PBL were obtained by centrifugation at 500× *g* for 30 min at 4 °C. The leukocytes were collected and washed twice with 10 mL PBS by centrifugation at 150× *g* for 10 min at 4 °C. All centrifugation was performed using a swinging bucket rotor centrifuge.

### 2.7. Flow Cytometry

Leukocytes (1 × 10^6^ cell/mL) from kidney, liver, spleen, and PBL from three olive flounders (62.3 ± 9.6 g at 16 °C) were prepared by centrifugation at 500 × *g* for 3 min according to Section 2.6. For flow cytometry analysis, leukocytes were incubated with respective CD marker mAbs- 4B2 (anti-CD3), 10F8-3 (anti-CD4-1) and 1D3 (anti-CD4-2) for 1 h at RT (room temperature, 25 °C), respectively [26,27,28]. This was followed by fluorescein isothiocyanate (FITC)-conjugated AffiniPure goat antimouse IgG (H + L; Jackson ImmunoResearch, Pennsylvania, USA) staining with a 1:200 dilution for 30 min at 37 °C. Negative controls were treated with FITC conjugate only. Leukocytes were washed twice with 1× PBS between each step, re-suspended in 1× PBS, and analyzed using a FACS Calibur™ (BD biosciences, San Jose, USA) flow cytometer, measuring at least 10,000 events per sample.

### 2.8. Measuring Population of CD3^+^, CD4-1^+^, and CD4-2^+^ T Cells in Olive Flounder after VHSV Infection and Vaccination

Fish challenged with VHSV or vaccinated, as described above, were observed for 28 days at 16 °C. Tissues were sampled from the kidney, liver, spleen, while blood was collected using heparinized syringes, as described in Section 2.6. Sampling was performed at 1, 3, 7, and 14 days post-infection (dpi) or days post-vaccination (dpv) in the VHSV infection group and the vaccinated fish, respectively. Leukocytes were isolated as described in Section 2.6. After isolating the leukocytes, cells were incubated with mAbs 4B2, 10F8-3, and 1D3 and the FITC-conjugated antibody as described in Section 2.7.

### 2.9. Stastical Analysis

The data are presented as the mean ± standard deviation (SD). Statistical *p* values of T cell population fold increase between the negative control and VHSV infection groups were calculated by Student’s *t*-test (*p* < 0.05) at each sampling time point. Significant differences (*p* < 0.05) in T cell population fold increase between the negative control and immune challenge groups, as well as between the FT-VHSV and Poly (I:C)-VHSV groups at each sampling time point were assessed using one-way ANOVA. All the statistical analysis was performed using SAS (Version 9.3, SAS Institute Inc., Cary, NC, USA) software.

## 3. Results

### 3.1. VHSV Challenge Test

**Figure 2:**
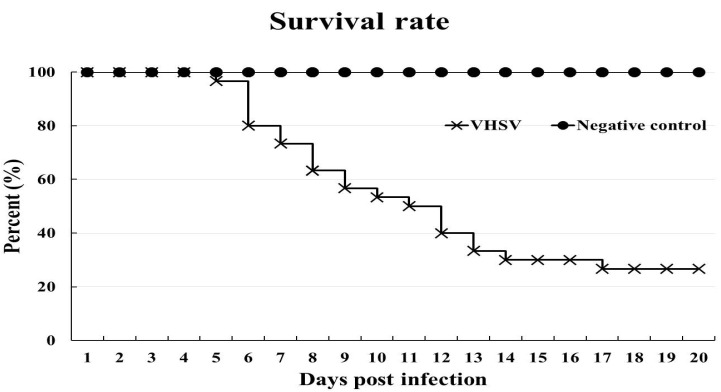
The survival rate of olive flounder after challenge with VHSV. Fish were intraperitoneally injected with VHSV (1 × 10^7^ TCID_50_/mL) in the VHSV infection group (*n* = 30). These fish had 26% survival while the negative control group (*n* = 30) had 100% survival.

**Figure 3:**
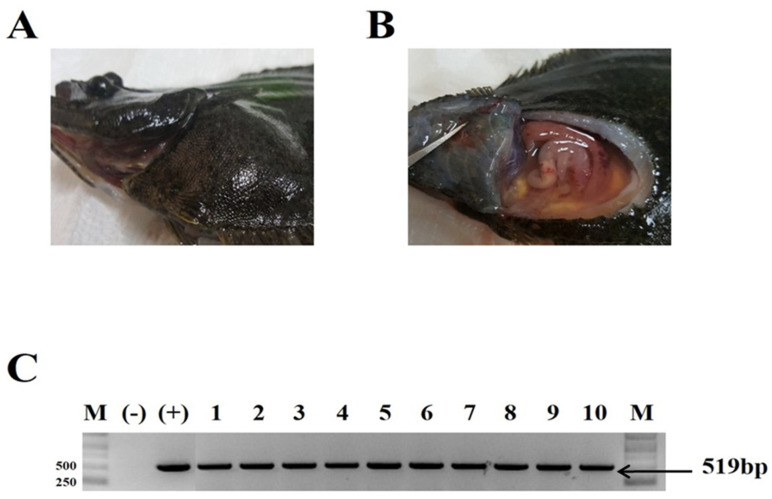
Clinical signs and molecular detection of VHSV in olive flounder. Gross signs of dead fish included skin darkening and a distended abdomen (**A**), the presence of ascites (**B**). Molecular detection of VHSV (type IV) glycoprotein gene (519 bp) from the spleen of dead fish (**C**). Molecular weight marker: M, base pair; Positive control: (+); Negative control: (-); Reverse transcripted cDNA from sampled fish using PCR amplicons (1–10)).

Fish in the VHSV infection group began to die from 5 dpi, with 26% of fish surviving (8 of 30 fish survived) at 20 dpi (when the challenge was terminated), while no fish in the negative control group died during the experiment (Figure 2). Confirmation of VHSV-related mortality was based on clinical signs and RT-PCR using spleen tissue from dead fish. Darkening of the skin (Figure 3A) and distended abdomen due to the large number of ascites (Figure 3B) observed. Spleens from ten olive flounders from the VHSV group served as a positive control for the VHSV RT-PCR. A fragment corresponding to 519 bp, as expected, was amplified in each challenged sample (Figure 3C). No bands were detected in the spleens taken from negative control fish.

### 3.2. Proliferation of T Cells Expressing CD3, CD4-1, and CD4-2 in Fish from the Experimental Infection

**Figure 4:**
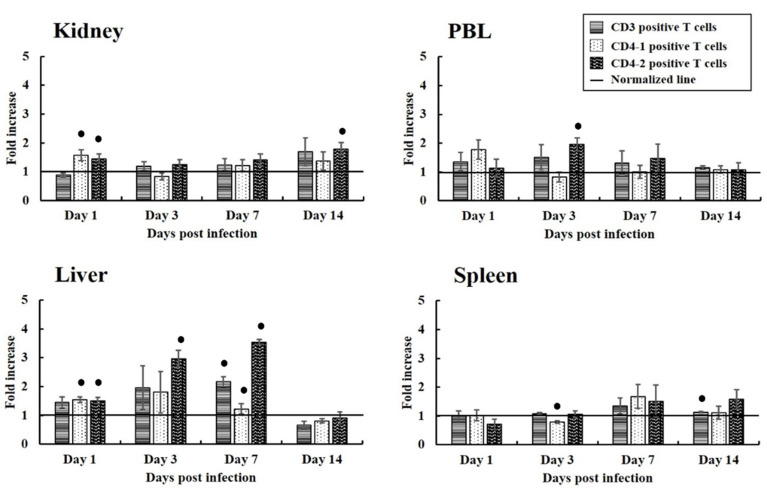
Fold increase in CD3^+^, CD4-1^+^, and CD4-2^+^ T cell populations during VHSV infection in olive flounder determined using flow cytometry. Leukocytes were isolated at 1, 3, 7, and 14 dpi from the kidney, liver, spleen and PBL (*n* = 3) from the VHSV infection (*n* = 30) and negative control (*n* = 30) groups, respectively. CD3^+^, CD4-1^+^, and CD4-2^+^ T cell responses were observed and presented as fold increase compared to the T cell population in the negative control group (adjusted to 1 and shown by a solid line). Error bars indicate the standard deviation of the mean. Black circles (●) represent a significant difference between the negative control and VHSV infection groups by Student’s *t*-test at a particular time point (*p* < 0.05).

Populations of CD3^+^, CD4-1^+^, and CD4-2^+^ T cells were identified by incubating leukocytes from the liver, spleen, kidney, and PBL with the respective mAbs, followed by flow cytometry analysis. Lymphocytes were gated on forward scatter-height (FSC-Height) and side scatter-height (SSC-Height) dot plots. The size of lymphocyte populations was expressed as the fold change relative to the average level of lymphocytes in the negative control group. The results obtained suggest that up-regulation of T cells depended on the progression of the viral infection. The levels of CD3^+^, CD4-1^+^, and CD4-2^+^ T cells in the liver, spleen, kidney, and PBL of infected fish differed over the course of infection (Figure 4). In the liver, CD3^+^ and CD4-2^+^ T cells gradually increased from 1 dpi to 7 dpi. CD3^+^ T cell showed a 1.46-fold increase at 1 dpi and statistically increased to a 2.17-fold by 7 dpi. Similarly, CD4-2^+^ T cells showed a 1.51-fold increase at 1 dpi then statistically increased to 3.54-fold by 7 dpi. Moreover, within the PBL T cell population, CD4-2^+^ T cells increased slightly, reaching a 1.96-fold increase statistically at 3 dpi.

### 3.3. Changes in Viral Titer due to Formalin Treatment and Survival Rate of Fish after Vaccination

The VHSV supernatant used in the vaccination studies had an initial infectivity dose of 1 × 10^7^ TCID_50_/mL. The infectivity titer of this decreased to 3.46 × 10^6^ TCID_50_/mL after storing for five days at 4 °C. This was injected into fish two days after treatment with Poly (I:C). In contrast, the titer of VHSV treated with formalin (0.1% *v*/*v*) dropped below the detection limit of the RT-PCR (10^2^ TCID_50_/mL) after five days exposure to formalin at 4 °C.

**Figure 5:**
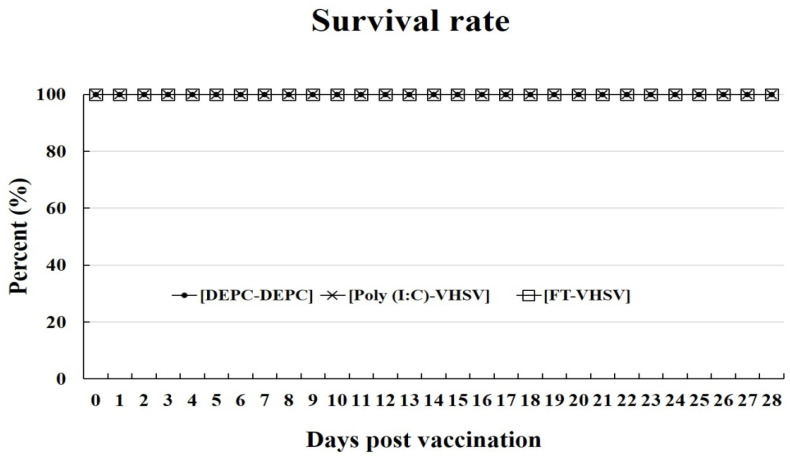
The level of survival in olive flounder after vaccination. Both Poly (I:C)-VHSV (*n* = 30) and FT-VHSV (*n* = 30) groups showed 100% survival after vaccination.

The survival rates of fish in the Poly (I:C)-VHSV and FT-VHSV groups were determined 28 dpv (Figure 5). No mortalities were recorded in any of the Poly (I:C)-VHSV, FT-VHSV, or negative control groups during the vaccination experiment.

### 3.4. Proliferation of T Cells Expressing CD3, CD4-1, and CD4-2 In Vivo after Vaccination

The expression levels of CD3^+^, CD4-1^+^, and CD4-2^+^ T cells in the liver, spleen, kidney. and PBL varied between the infection and vaccination trials over the course of each experiment. Changes in the levels of each mAb-positive T cell population in the different organs over the course of the vaccination trial in response to the different vaccines are presented below. The same mAbs and method described in Section 3.3 were used here, and the size of lymphocyte populations was again expressed as the fold change relative to the average number in the negative control group.

#### 3.4.1. CD3^+^ T Cells

**Figure 6:**
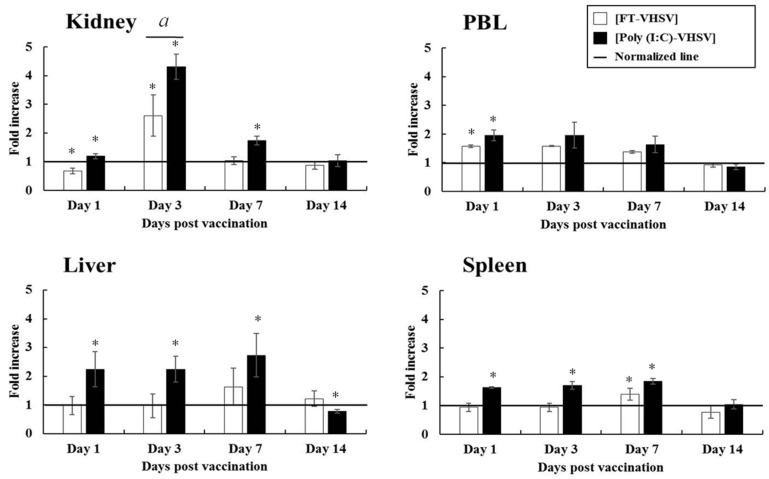
Fold increase in CD3^+^ T cell populations in olive flounder after poly (I:C) potentiated and formalin-treated VHSV vaccination determined by flow cytometry. Leukocytes were isolated at 1, 3, 7, and 14 dpv from the kidney, liver, spleen and PBL for three fish from both the Poly (I:C)-VHSV (*n* = 30) and the FT-VHSV (*n* = 30) groups. The CD3^+^ T cell response was observed and presented as a fold increase compared to the T cell population in the negative control group (adjusted to 1 and shown by a solid line). Error bars indicate the standard deviation of the mean. Asterisks (*) represent a significant difference between each experimental group and the negative control group by one-way ANOVA (*p* < 0.05). Symbol (*a*) indicates a statistical difference between the FT-VHSV and Poly (I:C)-VHSV groups by one-way ANOVA at a particular time point (*p* < 0.05).

The fold changes observed in CD3^+^ T cells in the liver, spleen, and PBL after poly (I:C)-potentiated vaccination increased from 1 dpv, and increased levels were maintained until 7 dpv, then decreased by 14 dpv. No difference was seen in the CD3^+^ T cell population in the kidney of the Poly (I:C)-VHSV group at 1 dpv, but a 4.30-fold increase was observed by 3 dpv, which was 1.65 times higher than seen in the FT-VHSV group. The fold increases in CD3^+^ T cells seen in the liver ranged from 2.25 to 2.63-fold between 1 dpv and 7 dpv in the Poly (I:C)-VHSV group compared to the 1.50-fold increase observed in the FT-VHSV group at 7 dpv (Figure 6).

#### 3.4.2. CD4-1^+^ T Cells

**Figure 7:**
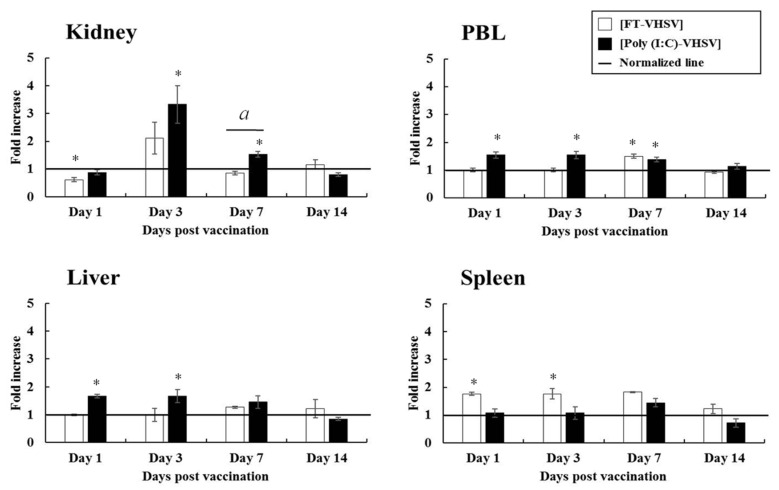
Fold increase in CD4-1^+^ T cell populations in olive flounder after poly (I:C) potentiated and formalin-treated VHSV vaccination determined by flow cytometry. Leukocytes were isolated at 1, 3, 7, and 14 dpv from the kidney, liver, spleen, and PBL for three fish from both the Poly (I:C)-VHSV (*n* = 30) and FT-VHSV (*n* = 30) groups. The CD4-1^+^ T cell response was observed and presented as a fold increase compared to the T cell population in the negative control group (adjusted to 1 and shown by a solid line). Error bars indicate the standard deviation of the mean. Asterisks (*) represent a significant difference between each experimental group and the negative control group by one-way ANOVA (*p* < 0.05). Symbol (*a*) indicates a statistical difference between the FT-VHSV and Poly (I:C)-VHSV groups by one-way ANOVA at a particular time point (*p* < 0.05).

In the Poly (I:C)-VHSV group, the distribution patterns of CD4-1^+^ T cells in the liver and PBL were similar to that of the CD3^+^ T cells but at lower levels. Significant increases in CD4-1^+^ T cells were seen in the kidney, wherein a 3.33-fold increase was obtained compared to non-vaccinated control at 3 dpv, and was 1.57 times higher than the FT-VHSV group. At 7 dpv, CD4-1^+^ T cells in the kidney increased by 1.53-fold, which was 1.91-fold greater compared to the FT-VHSV group. On the other hand, in the formalin-treated VHSV vaccination group, CD4-1^+^ T cells in the spleen showed a 1.76 to 1.80-fold change from 1 dpv to 7 dpv, while in the kidney, a 2.16-fold increase was observed (Figure 7).

#### 3.4.3. CD4-2^+^ T Cells

**Figure 8:**
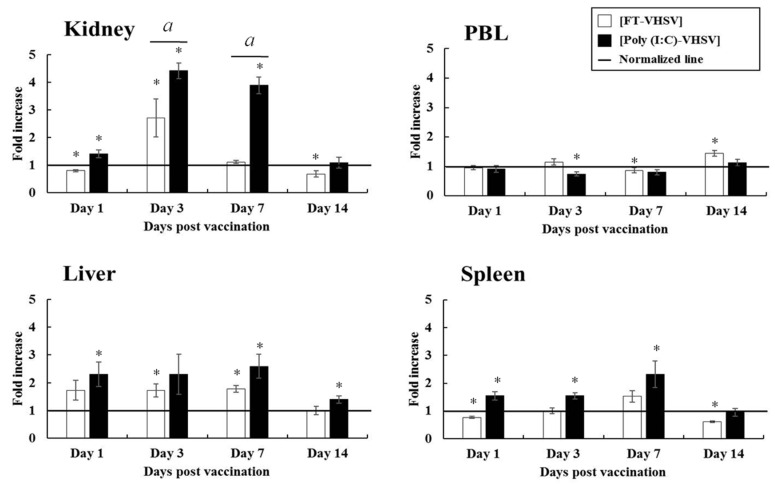
Fold increase in CD4-2^+^ T cell populations in olive flounder after poly (I:C) potentiated and formalin-treated VHSV vaccination determined by flow cytometry. Leukocytes were isolated at 1, 3, 7, and 14 dpv from the kidney, liver, spleen, and PBL for three fish from both the Poly (I:C)-VHSV (*n* = 30) and FT-VHSV (*n* = 30) groups. The CD4-2^+^ T cell response was observed and presented as a fold increase compared to the T cell population in the negative control group (adjusted to 1 and shown by a solid line). Error bars indicate the standard deviation of the mean. Asterisks (*) represent a significant difference between each experimental group and the negative control group by one-way ANOVA (*p* < 0.05). Symbol (*a*) indicates a statistical difference between the FT-VHSV and Poly (I:C)-VHSV group by one-way ANOVA at a particular time point (*p* < 0.05).

Except for PBL, all other tissues (liver, spleen, and kidney) showed a significant increase in CD4-2^+^ T cells (greater than CD4-1^+^ T cells) at 1 dpv in the Poly (I:C)-VHSV group. CD4-2^+^ T cells in the liver showed a 2.30 to 2.59-fold increase, whereas in the spleen, a 1.55 to 2.32-fold change was observed between 1 and 7 dpv. The most significant increase in CD4-2^+^ T cells was observed in the kidney, which was 4.42-fold higher than the negative control at 3 dpv, and it was 1.63-fold greater than the FT-VHSV group. At 7 dpv, the fold difference reached 3.89, which was 3.48 times higher than seen in the FT-VHSV group. On the other hand, CD4-2^+^ T cells in the livers of the FT-VHSV group showed a sustained 1.73-fold increase between 1 and 7 dpv, while in the spleen, the cells increased by 1.55-fold until 7 dpv (Figure 8).

## 4. Discussion

This study investigated T cell responses in olive flounder after infection with VHSV, or immune challenge with poly (I:C)-potentiated or formalin-treated VHSV vaccines, based on the distribution pattern of T cells in various organs of olive flounder after treatment. Levels of T cells apparently increased in all organs examined between 3 dpi to 7 dpi in the VHSV infection group. This reflects previous reports that show CD3^+^, CD4-1^+^, and CD4-2^+^ T cell populations normally peak in olive flounder between 3 dpi and 7 dpi after infection with VHSV [26,27,28]. The highest number of CD4-2^+^ T cells was observed in the liver at 7 dpi, which suggests direct involvement of virus-specific Th1 (CD4-2^+^) T cells during the peak of acute infection [40]. Although the liver-related immune response in teleost has not been investigated much yet, the hepatic immune of fish has recently been reporting including rainbow trout (*Oncorhynchus mykiss*), Arctic charr (*Salvelinus alpinus*), and olive flounder (*Paralichthys olivaceus*) [41,42,43,44,45,46]. Early infection with VHSV has been shown to increase transcription levels of some genes in the liver of rainbow trout, namely CD3 and CD4 [45], while CD4 and CD8 were seen to be increased in olive flounder [43]. Furthermore, intrahepatic immune cells (IHICs) act as resident antigen-presenting cells that prime naïve T cells in the early stage of viral infection in teleosts [47]. Our findings implicate the importance of the liver in mediating the cellular immune response of olive flounder against VHSV infection.

No mortality was observed in the Poly (I:C)-VHSV or FT-VHSV groups when the fish were challenged with VHSV, as expected since both vaccines were hypothesized to protect fish after VHSV infection. The difference between the VHSV infection and Poly (I:C)-VHSV groups was the pre-administration of poly (I:C), which we speculate to be the reason why 100% of fish survived despite being infected with the virus. The high level of survival recorded in this study corroborates the results of a previous study with olive flounder immunized with poly (I:C) and pathogenic VHSV [13,14].

Flow cytometry analysis performed on lymphocyte populations after vaccination revealed greater amounts of CD3^+^ T cell in Poly (I:C)-VHSV vaccine fish compared to the FT-VHSV group. Results also showed that the CD3^+^ T cells appeared earlier than other CD cell markers tested. Structurally, every mature T cell possesses CD3-TCR complexes [26]; thus, we speculate that poly (I:C)-potentiated vaccination efficiently initiated the stimulation of T cells. Moreover, CD4-2^+^ T cells were also produced earlier and in greater numbers than CD4-1^+^ T cells. The presence of CD 4-2^+^ T cells signifies their role in the antiviral immune response in olive flounder, triggered by the administration of poly (I:C). This has also been reported in mammals, wherein poly (I:C) has been used as an adjuvant to induced CD4-2^+^ T cell immunity [48].

Similarly, upregulation of immune-related genes such as Toll-like receptor 3 (TLR-3), Melanoma differentiation-associated protein 5 (Mda5), interferon 1 (IFN-1), and Mx protein has also been demonstrated in olive flounder within one day after poly (I:C) administration [49]. IFN-1 is a prominent antiviral cytokine directly stimulated by poly (I:C) that can create a virus-resistant state in the host [50]. Moreover, IFN-1 acts together with TLR-3 and Mda5 in promoting a T cell response that involves polarization of CD4^+^ T cells, producing Th1 (CD 4-2^+^ T cell) cells [51,52]. More importantly, the presence of viral particles (i.e., VHSV) along with poly (I:C) produces a synergistic effect on T cell immunity, thereby amplifying antiviral responses. Hence, this study strengthens our hypothesis that poly (I:C)-potentiated vaccination improves T cell immune response mediated through a synergistic effect between poly (I:C) and VHSV.

Poly (I:C)-potentiated vaccination was also seen to induce higher levels of CD4-2^+^ T cells in the kidney and spleen than seen with VHSV infection alone. The kidney and spleen are known to be primary and secondary lymphoid organs, respectively, in teleosts [52]. Since the thymus completely disappears seven months post-hatching in olive flounder, the kidney substitutes as the major hematopoietic and lymphoid and is responsible for the production of naïve T cells [53]. In addition to the kidney, the spleen also demonstrates hematopoietic functions in teleost [54,55,56]. We speculate that poly (I:C) induces transcription of IFN-1 followed by TLR-3 and Mda5 (from the hematopoietic cells), then finally leads to the differentiation of CD4^+^ T cells into CD4-2^+^ T cells (Th1) in the kidney and spleen [51].

Although the VHSV challenge was only conducted once, the level of survival in poly (I:C) vaccination group is consistently better than the other experimental groups. The observed antiviral effect of poly (I:C) is hypothesized to be caused by the sustained high levels of CD4-2^+^ T cells induced by poly (I:C) vaccination. CD4-2^+^ T cells have been reported to play an important role in generating effector CD8^+^ T cells and secreting essential cytokines, such as IFN-γ and IL-2 [57,58]. Effector CD8^+^ T cells, an important component of intracellular immunity (i.e., eliciting antiviral responses), are stimulated by IL-2 derived from activated CD4-2^+^ T cells [35,57]. Furthermore, fish IFN-γ includes several immune functions: (a) enhances phagocytic responses and host surveillance against viruses; (b) induces gene expression of many IFN-stimulated genes (ISGs), including Mx protein; (c) modulates cytokine and chemokine expression [59]. In mammals, IL-2 plays a key role in the activation of NK cells and effector CD8^+^ T cells, which are active components of cell-mediated immunity [57,60]. As a vital cytokine, IL-12 has also been studied in teleosts to determine its involvement in the immune defense mechanism against viral infections [60,61,62]. Homologs of IL-12 have already been reported in Japanese pufferfish (*Fugu rubripes*), rainbow trout (*Oncorhynchus mykiss*), large yellow croaker (*Larimichthyscrocea*), and olive flounder (*Paralichthys olivaceus*) [60,61,62,63]. In summary, our findings suggest that the protective effect of the poly (I:C)-potentiated vaccination in olive flounder is caused by the secretion of important cytokines that trigger cell-mediated immunity as represented by the increased levels of CD4-2^+^ T cells seen in our study.

## 5. Conclusions

The potency of poly (I:C)-potentiated vaccination was examined in this study through assessment of the fundamental immune cells, i.e., CD4-2^+^ T cells, involved in cellular immunity. Recognizing which arm of the immune system is initiated by poly (I:C)-potentiated vaccination provides important information on how this vaccination method could be utilized to its full potential as a preventive measure and/or treatment against viral infections in fish.
